# Single-channel seizure detection with clinical confirmation of seizure locations using CHB-MIT dataset

**DOI:** 10.3389/fneur.2024.1389731

**Published:** 2024-05-20

**Authors:** Yoon Gi Chung, Anna Cho, Hunmin Kim, Ki Joong Kim

**Affiliations:** ^1^Department of Pediatrics, Seoul National University Bundang Hospital, Seoul National University College of Medicine, Seongnam-si, Gyeonggi-do, Republic of Korea; ^2^Department of Pediatrics, Seoul National University College of Medicine, Seoul, Republic of Korea; ^3^Department of Pediatrics, Seoul National University Children’s Hospital, Seoul National University College of Medicine, Seoul, Republic of Korea

**Keywords:** deep learning, electroencephalography, epilepsy, seizure detection, single channel, wearable

## Abstract

**Introduction:**

Long-term electroencephalography (EEG) monitoring is advised to patients with refractory epilepsy who have a failure of anti-seizure medication and therapy. However, its real-life application is limited mainly due to the use of multiple EEG channels. We proposed a patient-specific deep learning-based single-channel seizure detection approach using the long-term scalp EEG recordings of the Children’s Hospital Boston-Massachusetts Institute of Technology (CHB-MIT) dataset, in conjunction with neurologists’ confirmation of spatial seizure characteristics of individual patients.

**Methods:**

We constructed 18-, 4-, and single-channel seizure detectors for 13 patients. Neurologists selected a specific channel among four channels, two close to the behind-the-ear and two at the forehead for each patient, after reviewing the patient’s distinctive seizure locations with seizure re-annotation.

**Results:**

Our multi- and single-channel detectors achieved an average sensitivity of 97.05–100%, false alarm rate of 0.22–0.40/h, and latency of 2.1–3.4 s for identification of seizures in continuous EEG recordings. The results demonstrated that seizure detection performance of our single-channel approach was comparable to that of our multi-channel ones.

**Discussion:**

We suggest that our single-channel approach in conjunction with clinical designation of the most prominent seizure locations has a high potential for wearable seizure detection on long-term EEG recordings for patients with refractory epilepsy.

## 1 Introduction

Epilepsy is a chronic neurological condition with a worldwide prevalence of 0.64% (1). The major symptom of epilepsy is seizures, and it is characterized by unprovoked and unexpected abnormal brain activity due to neuronal hyperexcitability and hypersynchrony (2). Approximately one-third of epileptic patients are suffering from refractory epilepsy (3, 4). Electroencephalography (EEG) is a critical initial step in the diagnosis of epilepsy owing to its powerful ability to uncover electrophysiological evidence of epileptic brain activity (5). As patients with refractory epilepsy tend to be exposed to injuries, psychosocial impairment, which seriously deteriorates their quality of life, or even death (6, 7), long-term EEG monitoring is essential for managing seizures and establishing appropriate treatment strategies (8–10). However, manual detection of seizures in long-term EEG recordings is highly time-consuming, labor-intensive, and clinician-dependent, and it often leads to misidentification and overmedication (11, 12).

Numerous automated seizure detection approaches based on machine and deep learning techniques have been proposed to overcome the burden of manual seizure detection (11, 13). Recently, many studies have adopted convolutional neural networks (CNN), recurrent neural networks (RNN), and hybrid CNN-RNN architectures for deep learning-based automated seizure detection approaches, with high performance in the identification of seizures on the scalp and intracranial EEG recordings. Despite the remarkable advancements in automated seizure detection, its real-life application for patients with refractory epilepsy is still limited because most approaches require multiple EEG channels, typically ≥18 for scalp EEG electrodes, to acquire sufficient amount of data in hospital environments. The use of multiple EEG electrodes makes patients uncomfortable and induces high computational complexity, particularly during long-term EEG monitoring.

Some studies on deep learning-based automated seizure detection have proposed various methods to reduce the number of EEG channels for patient-friendly and efficient machine-based examination of the occurrence of seizures on long-term scalp EEG recordings. They reported high seizure detection performance with reduced montage settings of at least two EEG channels, comparable to those with a full montage setting (14–23). In addition, some recent studies on machine (24–27) and deep learning-based (28) seizure detection approaches have utilized four behind-the-ear EEG channels. They demonstrated the feasibility of using a small number of channels for automated seizure detection in the long-term wearable EEG recordings of patients with refractory epilepsy. However, the approaches based on behind-the-ear channels are expected to mostly focused on seizures, predominantly in regions near the temporal lobes.

Seizure detection with single-channel EEG monitoring is the most convenient approach for patients who use wearable devices for long durations daily. Using one channel is expected to considerably enhance comfort and reduce complexity during long-term EEG monitoring. A recent study proposed a wearable approach for EEG acquisition using a device in the ear called ear-EEG and demonstrated its ability to detect electroencephalographic patterns of seizures in the temporal lobe (29). Other recent studies proposed a single-channel EEG sensor called Epilog, which could be easily attached to hairless regions such as the forehead or behind each ear (30, 31). They demonstrated that epileptologists and machine learning-based algorithms could competently identify focal seizures with single-channel EEG monitoring if the sensors were placed near the seizure onset foci. Based on these studies, using one channel is expected to guarantee automated identification of seizures during wearable EEG monitoring, at least for seizures near their locations on the scalp.

In this study, we proposed a deep learning-based patient-specific single-channel seizure detection approach in conjunction with clinical designation of the most prominent seizure locations. We built (1) 18-channel seizure detectors with a full-montage setting, (2) 4-channel detectors whose channel locations were fixed onto the forehead and behind both ears, and (3) single-channel detectors whose channels were selected based on neurologists’ confirmation of the spatial seizure characteristics of individual patients. Subsequently, we compared the seizure detection capabilities of single-channel detectors with those of multi-channel detectors to evaluate the practical usefulness of our single-channel approach for long-term wearable seizure detection.

## 2 Methods

### 2.1 Dataset

We used the Children’s Hospital Boston-Massachusetts Institute of Technology (CHB-MIT) scalp EEG database.^1^ This dataset consisted of scalp EEG recordings from 22 pediatric participants (5 males, aged 3–22, 17 females, aged 1.5–19). All EEG recordings were grouped into 23 cases from chb01 to chb23 (chb01 and chb21 were the same participants but chb21 was obtained 1.5 years later; chb24 was excluded from this study). Each case had 9–42 European data format (EDF) files sampled at 256 Hz with a 16-bit resolution. The EDF files had 1–4 recording hours with 23–26 channels, in accordance with the international 10–20 system. Herein, a total of 182 seizures were annotated in the publicly available EDF files of the 23 cases. As three EDF files of chb12 did not have longitudinal bipolar montages, their corresponding 13 seizures were excluded. In total, we obtained 169 seizures in the 23 cases. Each case represents an individual patient. The Institutional Review Board of Seoul National University Bundang Hospital approved this study (No. B-2205-758-105) and waived the requirement for informed consent due to the retrospective nature of the study. This study was conducted following the principles of the Declaration of Helsinki.

We selected 18 channels, namely Fp1-F3, F3-C3, C3-P3, P3-O1, Fp2-F4, F4-C4, C4-P4, P4-O2, Fp1-F7, F7-T7, T7-P7, P7-O1, Fp2-F8, F8-T8, T8-P8, P8-O2, Fz-Cz, and Cz-Pz, as a full montage setting. Subsequently, we selected four channels, Fp1-F3, Fp2-F4, P7-O1, and P8-O2, from the full montage setting. Two channels, P7-O1 and P8-O2, were selected because they were closest to the behind-the-ear positions. The other two channels, Fp1-F3 and Fp2-F4, were selected because they were placed on the forehead. These four channels were selected because they were expected to be the most common positions for wearable electrodes to be easily attached.

Two neurologists reviewed all seizures in the 23 cases to determine their distinctive locations representing spatial seizure characteristics on the scalp, such as the frontal, temporal, parietal, and occipital regions. As they reviewed only electrographic seizures without video data, seizure locations of six cases could not be identified (chb12, chb14, chb16, chb18, chb20, and chb21), and those of four cases were not close to Fp1-F3, Fp2-F4, P7-O1, or P8-O2 (chb06, chb09, chb13, and chb19). Therefore, they selected 13 cases whose seizure locations were identifiable by four or one of the four channels. Detailed information of the 13 cases is presented in Table 1.

**Table 1:** Patient information on the 13 cases of the CHB-MIT dataset.

Case	Age (yr)	Sex	Number of seizures	Seizure length (s)	Total seizure length (s)	Recording length (h)	Single-channel
chb01	11.0	F	7	50.6 ± 23.0	354	40.6	P8-O2
chb02	11.0	M	3	50.7 ± 36.3	152	35.3	P7-O1
chb03	14.0	F	7	39.3 ± 6.0	275	38.0	Fp1-F3
chb04	22.0	M	4	32.8 ± 16.3	131	156.1	P8-O2
chb05	7.0	F	5	71.4 ± 16.1	357	39.0	P7-O1
chb07	14.5	F	3	37.0 ± 7.5	111	67.1	Fp1-F3
chb08	3.5	M	5	42.4 ± 20.4	212	20.0	Fp1-F3
chb10	3.0	M	7	44.1 ± 14.6	309	50.0	P7-O1
chb11	12.0	F	3	46.7 ± 46.2	140	34.8	P7-O1
chb15	16.0	M	20	80.8 ± 46.5	1,616	40.0	P7-O1
chb17	12.0	F	3	78.0 ± 17.8	234	21.0	P8-O2
chb22	9.0	F	3	60.7 ± 11.0	182	31.0	Fp1-F3
chb23	6.0	F	7	49.4 ± 18.5	346	26.6	Fp1-F3
Total			77		4,419	599.5	
Mean ± SD	10.8 ± 5.3		5.9 ± 4.6	57.4 ± 32.9	339.9 ± 393.5	46.1 ± 35.2	

Seizure length represents mean ± standard deviation (SD) for our re-annotated seizures.

**Figure 1:**
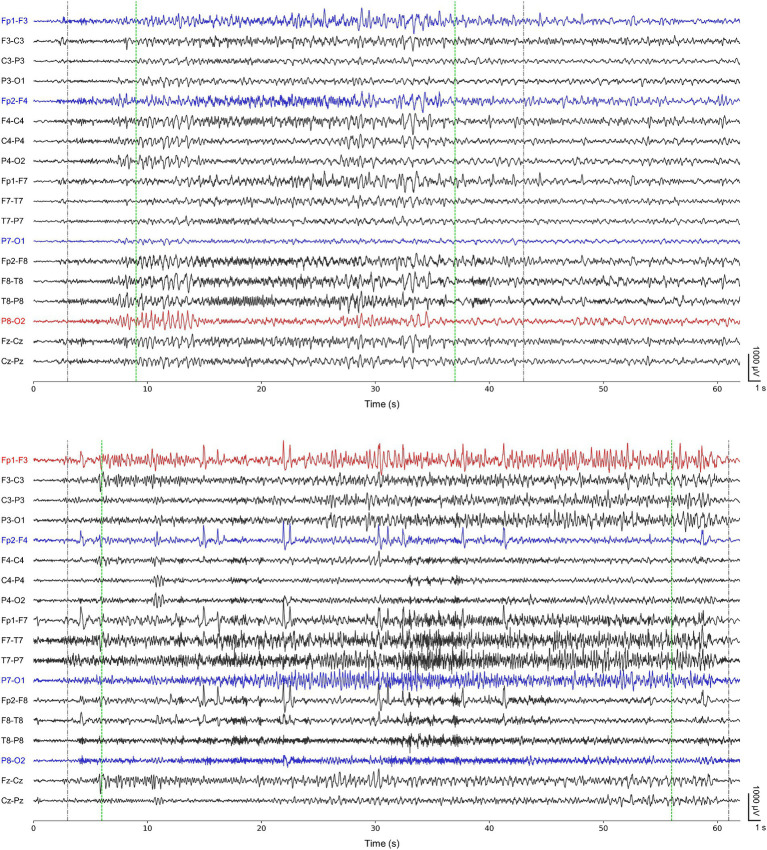
Representative ictal periods of the first seizure from chb01 (upper) and the first seizure from chb22 (lower). Gray-colored dotted lines represent publicly available annotations for onsets and terminations. Green-colored dotted lines represent our re-annotations for onsets and terminations. Both blue- and red-colored channels represent the channels for our 4-channel detectors. Red-colored channels represent P8-O2 and Fp1-F3 for single-channel detectors specific for chb01 and chb22, respectively.

Ictal period was defined as the interval between seizure onset and termination, and interictal period was defined as the remaining section after excluding all ictal periods. The neurologists re-annotated the ictal periods corresponding to 77 seizures of the 13 cases based on their confirmation of distinctive seizure locations to make the periods contain ictal EEG characteristics as clear as possible. They were requested to adjust the existing ictal periods (publicly available annotation) to have noticeable electrographic seizure-related EEG variations (re-annotation). Figure 1 shows representative ictal periods with publicly available annotations and our re-annotations.

### 2.2 Preprocessing

All EEG data were bandpass-filtered between 1 and 30 Hz as most rhythmic activities of seizures in scalp EEG recordings could be captured in this frequency band (32, 33). The bandpass-filtered EEG signals in the ictal and interictal periods were divided into 4-s long segments to analyze EEG oscillations from delta (1–4 Hz) to beta (13–30 Hz) bands. The EEG signals were split every one data point (1/256 s, approximately 4 ms) to maximize the number of segments, yielding an overlap of 3.996 s between two consecutive segments. EEG segments from the ictal and interictal periods were defined as the ictal and interictal segments, respectively. The number of interictal segments was much larger than that of ictal segments because the total length of the interictal periods (46.02 ± 35.26 h, averaged over 13 cases) was much longer than that of the ictal periods (0.09 ± 0.11 h). We used a balanced batch generator to create a subset of the dataset for each batch with the same number of randomly selected ictal and interictal segments during model training to handle the imbalanced dataset of ictal and interictal segments. No additional artifact rejection was performed to avoid signal distortion.

### 2.3 Patient-specific seizure detection

Similar to previous studies on deep learning-based seizure detection (34, 35), we applied *k*-fold cross-validation to train and evaluate our patient-specific seizure detectors, where *k* was the number of EDF files with at least one or more seizures in each case. First, we gathered interictal segments from all EDF files with no seizure. Next, we gathered ictal segments from *k*-1 EDF files with seizures. These datasets were used to construct a CNN-based binary classification model as a detector for classifying ictal and interictal segments, with a ratio of 7:2:1 for the training, validation, and test datasets (segment-level evaluation). Then, we used the remaining EDF file with seizures to evaluate seizure detection in the continuous EEG recordings. We applied the classification model trained using *k*-1 EDF files to the remaining EDF file to consecutively classify ictal and interictal segments based on the sliding-window technique using 4 s segments overlapping with a step size of 1 s (event-level evaluation). The length and number of seizures of each EDF file for the event-level evaluation varied from one to four and from one to five, respectively, depending on the case. We repeated both the segment- and event-level evaluation steps *k* times for each case.

In the segment-level evaluation, we designated an EEG segment with ictal probability greater than 0.5 as an ictal segment and less than 0.5 as an interictal segment. We used sensitivity, specificity, accuracy, and area under the receiver operating characteristic curve (AUC) as performance measures. We defined sensitivity, specificity, and accuracy as the number of correctly classified ictal segments divided by the number of true ictal segments, the number of correctly classified interictal segments divided by the number of true interictal segments, and the total number of correctly classified segments divided by the number of all test segments, respectively.

In the event-level evaluation, we performed three post-processing steps to acquire model-annotated seizures in the continuous EEG recordings. First, we used a Savitzky–Golay filter (36) with a window length of 10 and polynomial order of 3 to smooth time-varying ictal probability. Second, we collected segments with ictal probability exceeding a certain threshold *Th*. Third, we obtained *L* consecutive segments above the threshold. Any sets of *L* consecutive segments that were less than 10 s apart were merged. *Th* and *L* varied from 0.2 to 0.9 and from 5 to 10, respectively, depending on cases. If there were any overlaps between the model-annotated seizure and its nearby neurologist-annotated seizure, the model-annotated one was regarded as a correctly detected seizure. If no overlap, the neurologist-annotated one was regarded as a missed seizure. We defined a false alarm as a model-labeled seizure placed outside any neurologist-annotated seizures. We optimized the parameters of the Savitzky–Golay filter to minimize the number of false alarms. Further, we used sensitivity, false alarm rate (FAR), and latency as performance measures. We defined sensitivity, FAR, and latency as the number of correctly detected seizures divided by the number of neurologist-annotated seizures, number of false alarms divided by the length of the test EEG recording, and the time delay between the onsets of the model- and neurologist-annotated seizures, respectively.

**Figure 2:**
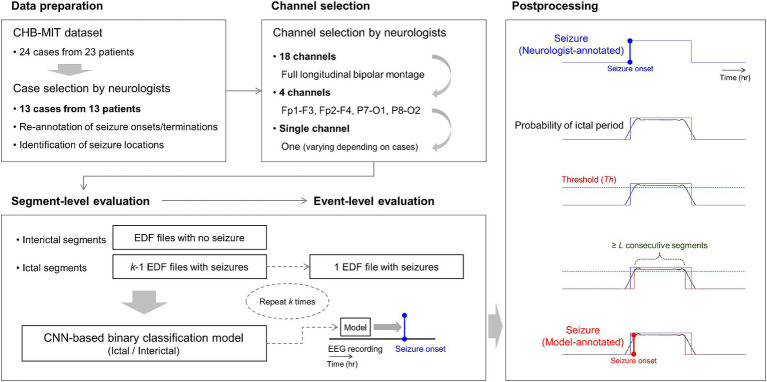
Overall process of our 18-, 4-, and single-channel seizure detection. Neurologists select one specific channel among the four channels of Fp1-F3, Fp2-F4, P7-O1, and P8-O2 for each patient based on the identification of seizure locations with re-annotation. *k*-fold cross-validation is applied to segment- and event-level evaluation steps, where *k* is the number of European data format files with at least one or more seizures in each patient. In the figure at the bottom of postprocessing, the blue-colored line represents a neurologist-annotated seizure. The red-colored line represents a model-annotated seizure overlapped with the neurologist-annotated seizure, indicating a correctly detected seizure. CNN denotes convolutional neural network.

To construct 18-channel detectors, we used 18-channel EEG time series at the full montage setting as input data for the classification models. To construct 4-channel detectors, we used 4-channel EEG time series from Fp1-F3, Fp2-F4, P7-O1, and P8-O2 as input data for the classification models. To construct single-channel detectors, our neurologists selected one channel based on their confirmation of distinctive seizure locations for each case. They were requested to select one specific channel from the abovementioned four channels. For example, they selected Fp1-F3 if they determined the left-side frontal region to be the most distinctive. Subsequently, we used the single-channel EEG time series as input data for the classification models. Detailed information on the overall process is presented in Figure 2.

### 2.4 CNN architecture

**Figure 3:**
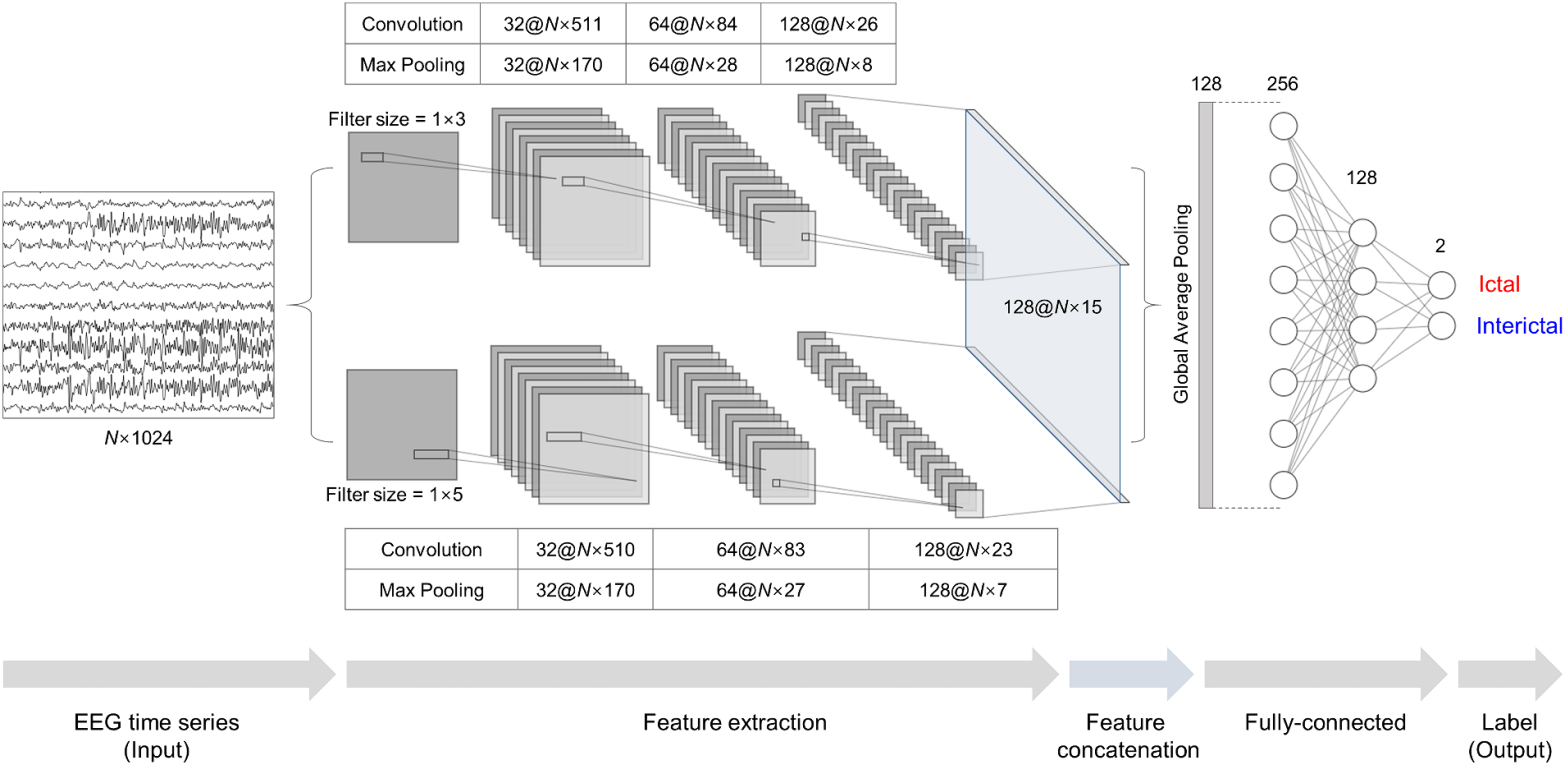
Stacked two-dimensional (2D) convolutional neural network (CNN) architecture for ictal-interictal binary classification. Multi- or single-channel EEG time series with a size of *N* × 1,024 are fed into two 2D CNN modules individually as input data. The two 2D CNN modules extract features using filters with a size of 1 × 3 and 1 × 5. The features are concatenated and fed into fully-connected layers for outputs using a softmax function.

We adopted a stacked two-dimensional (2D) CNN architecture, similar to the one-dimensional (1D) architecture used in the previous study (35), for our binary classification models. We constructed two 2D CNN modules in parallel. Each module consisted of three convolution, batch normalization, and max-pooling layers. As we used multi- or single-channel EEG time series as input, 4 s EEG segments with a size of *N* × 1,024 were fed into the input layer, where *N* was the number of channels. The convolution layers contained 32, 64, and 128 filters with rectified linear units as their activation functions. One module had a filter size of 1 × 3, and the other one had a filter size of 1 × 5 with strides of 2, 2, and 1 for three convolution layers. Max-pooling layers had a pooling size of 3. The final layers of the two modules were concatenated into a single feature layer. The concatenated feature vector was flattened by global average pooling and fed into fully connected layers. We obtained an output vector using a softmax function from the fully connected layers with ictal probability ranging from zero to one. An input segment was classified into an ictal one if its ictal probability was ≥0.5 and interictal one if <0.5. We used root mean square propagation with a learning rate of 3 × 10^−4^ as an optimizer, binary cross-entropy as a loss function, and early stopping with a patience of 15 to avoid overfitting. We used Python 3.8, TensorFlow 2.2, compute unified device architecture (CUDA) 10.1, and four NVIDIA TITAN V graphic cards with 12 GB of memory. Detailed information on the CNN architecture is presented in Figure 3.

## 3 Results

### 3.1 18-channel seizure detection

We observed the ictal-interictal binary classification performance of our patient-specific 18-channel seizure detectors in the segment-level evaluation, with a sensitivity, specificity, accuracy, and AUC of 98.66 ± 1.19% (mean ± standard deviation), 98.47 ± 2.71%, 98.47 ± 2.69%, and 98.56 ± 1.81%, respectively, averaged over the 13 cases. Among the 13 cases, chb02 and chb04 exhibited the highest accuracy of 99.97 ± 0.03% and 99.97 ± 0.02%, respectively (sensitivity and specificity of 98.12 ± 3.25% and 99.97 ± 0.03%; 98.18 ± 2.52% and 99.97 ± 0.02%, respectively). However, chb15 exhibited the lowest accuracy of 90.33 ± 8.93% (96.20 ± 5.32% and 90.24 ± 9.13%, respectively), averaged over *k*-fold cross-validation.

We observed the seizure-detection performance of the 18-channel detectors in the event-level evaluation with a sensitivity, FAR, and latency of 100%, 0.30 ± 0.47/h, and 2.1 ± 6.7 s, averaged over the 13 cases, respectively. The 18-channel seizure detection exhibited a mean number of correctly detected seizures, missed seizures, and false alarms of 5.9 ± 4.6, 0, and 2.4 ± 4.4, respectively, during a mean length of EEG recordings of 7.0 ± 4.1 h. All patients exhibited 100% sensitivity. However, chb11 and chb15 exhibited FAR of 1.43/h and 1.14/h, respectively, thus indicating more than one false alarm per hour.

### 3.2 4-channel seizure detection

The ictal-interictal binary classification performance of our patient-specific 4-channel seizure detectors in the segment-level evaluation had a sensitivity, specificity, accuracy, and AUC of 97.31 ± 3.78%, 97.72 ± 3.61%, 97.73 ± 3.59%, and 97.51 ± 2.94%, respectively, averaged over the 13 cases. Among the 13 cases, chb04 and chb05 exhibited the highest accuracy of 99.92 ± 0.07% and 99.92 ± 0.06%, respectively (sensitivity and specificity of 93.75 ± 10.83% and 99.92 ± 0.07%; 99.69 ± 0.70% and 99.92 ± 0.06%, respectively). However, chb15 exhibited the lowest accuracy of 87.93 ± 10.51% (sensitivity and specificity of 92.80 ± 9.47% and 87.86 ± 10.79%, respectively) averaged over *k*-fold cross-validation.

We observed the seizure detection performance of the 4-channel detectors in the event-level evaluation with a sensitivity, FAR, and latency of 97.05 ± 9.23%, 0.40 ± 0.77/h, and 3.5 ± 4.8 s, averaged over the 13 cases. The 4-channel seizure detection exhibited a mean number of correctly detected seizures, missed seizures, and false alarms of 5.8 ± 4.4, 0.2 ± 0.4, and 2.9 ± 6.1, respectively, during a mean length of EEG recordings of 7.0 ± 4.1 h. Among the 13 cases, chb11 and chb15 exhibited a FAR of 2.51/h and 1.57/h, respectively, whereas chb17 exhibited the lowest sensitivity (66.67%) with no false alarm.

### 3.3 Single-channel seizure detection

Among the channels in our 4-channel seizure detection approach, the left frontal one (Fp1-F3) was assigned to five cases of chb03, chb07, chb08, chb22, and chb23; the left parieto-occipital one (P7-O1) to five cases of chb02, chb05, chb10, chb11, and chb15; and the right parieto-occipital one (P8-O2) to three cases of chb01, chb04, and chb17. No cases were assigned to the right frontal channel (Fp2-F4).

The ictal-interictal binary classification performance of our patient-specific single-channel seizure detectors in the segment-level evaluation had a sensitivity, specificity, accuracy, and AUC of 96.76 ± 3.97%, 98.19 ± 1.82%, 98.18 ± 1.83%, and 97.47 ± 2.77%, respectively, averaged over the 13 cases. Among the 13 cases, chb02 and chb11 exhibited the highest accuracy of 99.94 ± 0.02% (sensitivity and specificity of 99.75 ± 0.44% and 99.94 ± 0.02%; 100% and 99.94 ± 0.02%, respectively), whereas chb23 exhibited the lowest accuracy of 93.92 ± 8.54% (sensitivity and specificity of 87.49 ± 21.67% and 93.96 ± 8.61%, respectively) averaged over *k*-fold cross-validation.

The seizure detection performance of the single-channel detectors in the event-level evaluation had a sensitivity, FAR, and latency of 99.62 ± 1.39%, 0.22 ± 0.34/h, and 3.3 ± 5.5 s, averaged over the 13 cases, respectively. Single-channel seizure detection exhibited a mean number of correctly detected seizures, missed seizures, and false alarms of 5.8 ± 4.3, 0.1 ± 0.3, and 1.0 ± 1.2, respectively, during a mean length of EEG recordings of 7.0 ± 4.1 h. None of the cases exhibited an FAR higher than 1/h. Among the 13 cases, chb02 exhibited the highest FAR of 0.88/h with a sensitivity of 100%, whereas chb15 exhibited the lowest sensitivity (95%) with an FAR of 0.07/h.

Additionally, we performed single-channel seizure detection based on the publicly available annotations, to explore substantial effects of our re-annotation on the performance. The ictal-interictal binary classification performance of our patient-specific single-channel seizure detectors trained using publicly available annotations in the segment-level evaluation had a sensitivity, specificity, accuracy, and AUC of 96.39 ± 2.75%, 94.92 ± 8.38%, 94.93 ± 8.35%, and 95.62 ± 4.20%, respectively, averaged over the 13 cases. Among the 13 cases, chb02 exhibited the highest accuracy of 99.84 ± 0.12% (sensitivity and specificity of 100% and 99.84 ± 0.12%, respectively), whereas chb17 exhibited the lowest accuracy of 70.10 ± 10.86% (sensitivity and specificity of 100% and 70.01 ± 10.88%, respectively), averaged over *k*-fold cross-validation.

The seizure detection performance of the single-channel detectors trained by the publicly available annotations in the event-level evaluation had a sensitivity, FAR, and latency of 97.69 ± 6.96%, 0.16 ± 0.26/h, and 8.0 ± 9.4 s, averaged over the 13 cases, respectively. Single-channel seizure detection based on publicly available annotations exhibited a mean number of correctly detected seizures, missed seizures, and false alarms of 5.8 ± 4.4, 0.2 ± 0.4, and 1.2 ± 2.1, respectively, during a mean length of EEG recordings of 7.0 ± 4.1 h. None of the cases exhibited an FAR higher than 1/h. Among the 13 cases, chb02 exhibited the highest FAR of 0.88/h with a sensitivity of 100%, whereas chb04 exhibited the lowest sensitivity (75%) with an FAR of 0.09/h. Detailed results of the performances of our 18-, 4-, and single-channel seizure detectors averaged over the 13 cases are presented in Table 2 and Supplementary Tables S1–S4.

**Table 2:** Performance of patient-specific seizure detectors for ictal-interictal classification (segment-level) and seizure detection (event-level) averaged over the 13 cases by mean ± standard deviation.

	Segment-level evaluation				Event-level evaluation					
Detector	Sensitivity (%)	Specificity (%)	Accuracy (%)	AUC (%)	Sensitivity (%)	FAR (/h)	Latency (s)	Correctly detected seizure	Missed seizure	False alarm
18-channel	98.66 ± 1.19	98.47 ± 2.71	98.47 ± 2.69	98.56 ± 1.81	100.00 ± 0.00	0.30 ± 0.47	2.1 ± 6.7	5.9 ± 4.6	0.0 ± 0.0	2.4 ± 4.4
4-channel	97.31 ± 3.78	97.72 ± 3.61	97.73 ± 3.59	97.51 ± 2.94	97.05 ± 9.23	0.40 ± 0.77	3.5 ± 4.8	5.8 ± 4.4	0.2 ± 0.4	2.9 ± 6.1
Single-channel	96.76 ± 3.97	98.19 ± 1.82	98.18 ± 1.83	97.47 ± 2.77	99.62 ± 1.39	0.22 ± 0.34	3.3 ± 5.5	5.8 ± 4.3	0.1 ± 0.3	1.0 ± 1.2
Single-channel (Publicly available annotation)	96.39 ± 2.75	94.92 ± 8.38	94.93 ± 8.35	95.62 ± 4.20	97.69 ± 6.96	0.16 ± 0.26	8.0 ± 9.4	5.8 ± 4.4	0.2 ± 0.4	1.2 ± 2.1

An average EEG length for event-level evaluation is 7.0 ± 4.1 h. AUC and FAR denote area under the receiver operating characteristic curve and false alarm rate, respectively.

## 4 Discussion

We conducted patient-specific deep learning-based automated seizure detection in long-term scalp EEG recordings by reducing the number of channels from 18, 4 to 1, yielding a sensitivity of 97.05–100%, FAR of 0.22–0.40/h, and latency of 2.1–3.4 s averaged over the 13 cases of the CHB-MIT dataset. We obtained 18-channel seizure detectors with a full montage setting, 4-channel ones with two channels close to behind-the-ear positions and two channels at the forehead, and single-channel detectors with one specific channel among the four channels designated by the neurologists’ confirmation of spatial seizure characteristics for individual patients. We examined that our single-channel detectors successfully identified seizures in long-term scalp EEG recordings comparable to our multi-channel detectors, thus suggesting the usefulness of our single-channel approach for wearable seizure detection in conjunction with neurologists’ clinical designation of the most prominent seizure locations and its corresponding channel selection.

### 4.1 Previous seizure detection approach

Numerous studies have been suggested patient-specific deep learning-based automated seizure detection approaches using the CHB-MIT dataset (21, 34, 35, 37–48). In terms of seizure detection with a full montage setting, Xu et al. (44) proposed seizure detectors based on a three-dimensional (3D) CNN using time-frequency matrices by multiscale short-time Fourier transform with an average sensitivity, FAR, and latency of 94.95%, 0.08/h, and 2.3 s, respectively. Tang et al. (42) proposed a bidirectional long short-term memory (LSTM) network based on the attention mechanism and path signature algorithm yielding a seizure classification accuracy of 99.09%. Zhao et al. (48) proposed a hybrid attention network with graph modes and transformers yielding a seizure classification accuracy of 98.30%. Zhang et al. (47) proposed seizure detectors based on bidirectional gated recurrent units (GRUs) using time series decomposed by wavelet analysis, which exhibited an average sensitivity and FAR of 95.49% and 0.31/h, respectively. Wang et al. (35) proposed seizure detectors based on a stacked 1D CNN using multi-channel time series, which exhibited an average sensitivity, FAR, and latency of 99.31%, 0.20/h, and 8.1 s, respectively. Li et al. (41) proposed seizure detectors based on a CNN-LSTM hybrid architecture using multi-channel time series, which exhibited an average sensitivity and FAR of 95.29% and 0.66/h, respectively.

Despite the increase in the number of studies on full-montage seizure detectors, automated seizure detection with a reduced number of channels using the CHB-MIT dataset has been performed primarily using conventional machine learning techniques. Song et al. (49) proposed a channel screening method based on the refine composite multiscale dispersion entropy with residual convolutional LSTM yielding a seizure classification accuracy of 96.49% averaged over 14 subjects. However, this study had no result on the event-level evaluation. Tang et al. (21) proposed seizure detectors with five channels based on an autoencoder and support vector machine (SVM), yielding an average sensitivity, FAR, and latency of 97.2%, 0.64/h, and 1.1 s, respectively. Khanmohammadi et al. (40) proposed seizure detectors with five channels based on adaptive distancing, yielding an average sensitivity, FAR, and latency of 96%, 0.12/h, and 4.2 s, respectively. In the aforementioned studies, the channels were automatically selected using mathematical algorithms with amplitude variations or channel correlations. Rather than using channel-selection algorithms, a recent study manually selected specific channels that were expected to be important for seizure detection. Asif et al. (37) offered seizure detectors with 6–12 channels near the temporal lobe based on the random undersampling and boosting (RUSBoost), yielding an average sensitivity, FAR, and latency of 92%, 0.21/h, and 7.1 s, respectively, for their 10-channel detectors. They demonstrated the feasibility of using seizure detectors with a small number of channels in patients with temporal lobe epilepsy. In addition, they reported the performance of their 23-channel detectors with an average sensitivity, FAR, and latency of 95%, 0.16/h, and 6.8 s, respectively, suggesting an acceptable performance loss by channel reduction. Detailed information on the performance of previous studies using the CHB-MIT dataset for the event-level evaluation is presented in Table 3. Note that, in this study, our neurologists selected only one channel for single-channel seizure detection for each patient compared with other studies using less than full channel.

**Table 3:** Seizure detection performance of previous studies using the CHB-MIT dataset in comparison with ours.

Study	Number of channels	Input data	Deep/machine learning	Sensitivity (%)	FAR (/h)	Latency (s)
Y. Xu et al.	*Full*	Multiscale STFT	3D-CNN	94.95	0.08	2.3
Y. Zhang et al.	*Full*	Time series decomposition by DWT	Bi-GRU GRU	95.49 (Bi-GRU) 92.96 (GRU)	0.31 0.57	
X. Wang et al.	*Full*	Time series	Stacked 1D-CNN	99.31	0.20	8.1
Y. Li et al.	*Full*	Time series	CNN-LSTM	95.29	0.66	
Y. Guo et al.	*Full*	Time series decomposition by DWT	EasyEnsemble	97.50 ~ 100	0.91 ~ 1.38	
R. Zanetti et al.	*Full*	Approximate zero-crossing	Random forest	77.27	0.09	
L. S. Vidyaratne et al.	*Full*	Fractal dimension estimation Harmonic wavelet packet transform	Relevance vector machine	96.00	0.10	1.9
M. Zabihi et al.	*Full*	Phase space reconstruction	LDA and Naive Bayes	91.34 ~ 96.29	3.04 ~ 4.86	4.65 ~ 5.03
B. Hunyadi et al.	*Full*	Feature-channel matrix	SVM	80 ~ 83	0.41 ~ 0.88	8.5 ~ 10.0
A. Shoeb et al.	*Full*	Time series decomposition by DWT	SVM	96.00	0.08	Among 173 seizures, <3 s (50%) <5 s (71%) <10 s (91%)
F. -G. Tang et al.	5	Time series decomposition by DWT Frequency-domain features by autoencoder	SVM	97.20	0.64	1.1
R. Asif et al.	6, 8, 10, and 12 on temporal region	Time-domain statistical features	RUSBoost	95 (full channels) 92 (10 channels)	0.16 0.21	6.83 7.1
S. Khanmohammadi et al.	5	Time domain statistical features Spectral power	Adaptive distance-based change point detector	96.00	0.12	4.2
This study	18	Time series	Stacked 2D-CNN	100.00	0.30	2.1
	4	Time series	Stacked 2D-CNN	97.05	0.40	3.4
	Single	Time series	Stacked 2D-CNN	99.62	0.22	3.3

*Full* in the column of number of channels represents ≥18 channels. STFT and DWT in the column of input data denote short-time Fourier transform and discrete wavelet transform, respectively. CNN, GRU, LSTM, LDA, SVM, and RUSBoost denote convolutional neural network, gated recurrent unit, long short-term memory, linear discriminant analysis, support vector machine, and random undersampling and boosting, respectively. FAR denotes false alarm rate.

### 4.2 Proposed seizure detection approach

In this study, we built 18-channel detectors with a full montage setting that covered the entire scalp; 4-channel detectors with the smallest number of channels that could cover the frontal, temporal, parietal, and occipital regions; and single-channel detectors with one channel that could cover specific local area showing distinctive seizure characteristics. For our 4-channel detectors, the neurologists selected two channels, P7-O1 and P8-O2, which were closest to the behind-the-ear positions among the 18 channels in the international 10–20 system. They selected two additional channels, Fp1-F3 and Fp2-F4, close to the forehead to detect seizures occurring in the frontal region. We had several reasons for the selection of the four channels as follows: first, behind-the-ear EEG monitoring was a well-known wearable approach, considering temporal lobe seizures known as the most common focal ones (26, 28, 50); second, it could be challenging for the behind-the-ear monitoring to cover frontal regions; and third, both the behind-the-ear and forehead positions were expected to be suitable for easily attaching electrodes owing to minimal amount of hair. In terms of our single-channel approach, we can consider a situation in which neurologists provide single-channel seizure detection systems to patients with refractory epilepsy after confirming the most critical seizure-generating regions on the patient’s scalp. Thus, patients can use their systems comfortably with only one channel for a long period. No channel selection algorithm was used for our 4- and single-channel detectors because we intended to adopt only the neurologists’ clinical opinions. We observed that the performance of our single-channel seizure detector was comparable to that of other studies, as presented in Table 3. Channel selection methods of previous studies using the CHB-MIT dataset in comparison with our single-channel seizure detection approach is shown in Supplementary Table S5.

**Figure 4:**
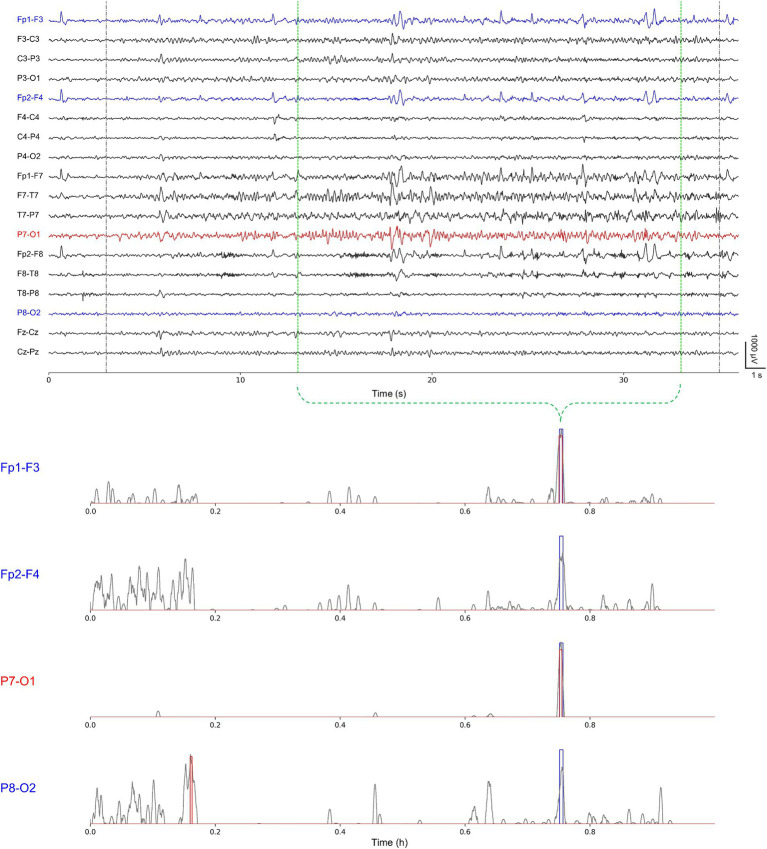
A representative single-channel seizure detection based on our event-level evaluation for the second seizure of chb11 using Fp1-F3, Fp2-F4, P7-O1, and P8-O2 individually. Gray-colored dotted lines represent publicly available annotations for onsets and terminations; green-colored dotted lines represent our re-annotations for onsets and terminations (upper). Gray-colored solid lines represent ictal probabilities; blue-colored rectangles represent neurologist-annotated seizures; and red-colored rectangles represent model-annotated seizures (lower).

Overall, we confirmed that no significant difference existed in detection abilities with respect to the number of channels. However, we observed that, in some cases, the number of channels had some influence on the seizure detection performance in the event-level evaluation. We examined the cases having differences in the false alarms ≥2 and the latency ≥3 s. Two cases of chb02 and chb08 showed an increase, whereas four cases of chb03, chb10, chb11, and chb15 showed a decrease in the number of false alarms for single-channel seizure detection compared with multi-channel one. Two cases of chb05 and chb17 showed an increase, whereas chb15 showed a decrease in the latency for single-channel seizure detection compared with multi-channel one. In particular, chb15 showed remarkable increases in the performance in terms of both the false alarms and latency. We suggest that single-channel seizure detection can exclude artifact-contaminated or unnecessary channels, possibly resulting in the reduction of the number of false alarms and the latency using less contaminated training datasets. However, there may be any loss of information on inter-channel relationships which can affect seizure detection ability. Therefore, further studies on how spatial epileptic EEG features vary in accordance with the number of channels in individual cases. Figure 4 shows a representative single-channel seizure detection based on our event-level evaluation for the second seizure of chb11. It describes that our single-channel detector using P7-O1 designated by the neurologists as the most prominent seizure location provides the best performance compared with others. That using Fp1-F3 also detects the seizure, but it generates more ictal probabilities potentially inducing false alarms. Single-channel seizure detection performance of chb11 in accordance with four channels is shown in Supplementary Table S6.

### 4.3 Wearable seizure detection

Recent studies have proposed patient-specific automated wearable seizure detection approaches using 4-channel behind-the-ear scalp EEG recordings with deep learning and machine learning techniques to achieve real-time identification of seizures in long-term EEG recordings. You et al. (28) reported an average sensitivity and FAR of 94.2% and 0.29/h, respectively, for their variational autoencoder-based approaches. Zhang et al. (27) reported an average sensitivity and FAR of 87.5% and 1.93/h, respectively; Swinnen et al. (25) reported 98.3% and 0.91/h, respectively; Vandecasteele et al. (26) reported 69.1% and 0.02/h, respectively; and Gu et al. (24) reported 94.5% and 0.52/h, respectively, for their SVM-based approaches. They suggested that behind-the-ear EEG monitoring was highly convenient, particularly for patients requiring continuously examination in out-of-hospital settings. However, we were concerned that seizure-detection approaches using the behind-the-ear channels could be mostly focused on the seizures occurring near the temporal lobe. Other recent studies utilized a single-channel EEG sensor called Epilog for the feasibility of long-term wearable seizure detection outside the hospital. Frankel et al. (31) demonstrated that epileptologists could successfully identify seizures in single-channel EEG recordings when the sensors were placed near the seizure onset foci with an accuracy higher than 80%. They (30) also reported a random forest-based seizure detection approach using single-channel EEG recordings with an average sensitivity and FAR of 87.5% and 0.14/h, respectively.

As we used the CHB-MIT dataset, comparing the performance of our study with that of the aforementioned studies was challenging. However, in terms of wearable seizure detection, we believe that this study has two key strengths compared with the previous ones as follows: first, neurologists select the most prominent seizure location for each patient from the 18-channel longitudinal bipolar montage in accordance with the international 10–20 system after reviewing electroencephalographic seizure signatures directly; second, our single-channel approach is expected to be applicable to both focal and generalized seizures due to the use of the aforementioned neurologist-selected channel for each patient regardless of the seizure types.

### 4.4 Seizure re-annotation

Instead of using publicly available annotations in the CHB-MIT dataset as in previous studies, we modified the annotations based on our neurologists’ re-examination of seizure onsets and terminations for the following two reasons. First, we needed to determine which region of the scalp had the most distinct seizure characteristics and which channel could be the most important for single-channel seizure detection in individual patients. For this purpose, we shifted the positions of seizure onsets and terminations to disclose spatial seizure characteristics as clearly as possible. Second, we intended to obtain training data for our classification models that were less contaminated than those from publicly available annotations. We assumed that our single-channel detectors would be stable for unseen data, which was in line with a previous study on the robustness of deep neural networks with a sufficient volume of cleanly labeled data (51).

In our results, chb17 showed a remarkable increase of >26% on average in the specificity of its classification models based on our re-annotations. In the event-level evaluation, six cases of chb04, chb05, chb07, chb08, chb15, and chb22 showed a decrease in the latency (≥3 s). Additionally, two cases of chb10 and chb15 exhibited a decrease in the false alarms (≥2). Particularly, chb15 exhibited a marked reduction in the number of false alarms using our re-annotations. We suggest that our re-annotations will benefit some cases, particularly those with considerably high latency and false alarms in single-channel seizure detection. Similarly, a previous study reported significant performance enhancement in wearable EEG-based seizure detection with automatic annotation correction (27). However, two cases of chb08 and chb11 showed an increase in the false alarms (≥2). Further studies are required to understand the possible negative effects of our re-annotations on seizure detection.

### 4.5 Limitations and future research

This study has some limitations. First, we used patient-specific seizure detection approaches. Numerous recent studies on patient-independent seizure detection approaches have shown high performance with sensitivity >85% and FAR<0.5/h with a full montage setting using the CHB-MIT dataset (52–54). A patient-independent approach is required to establish the generalizability of our single-channel seizure detection. Second, we adopted a 2D CNN architecture with two 2D CNN modules stacked in parallel to efficiently extract time-varying electroencephalographic signatures using two types of filter lengths, similar to the previous study on stacked 1D CNN-based seizure detection (35). However, other studies have adopted other deep-learning methods, such as 3D CNNs (44), transformers (48), GRUs (47), and a hybrid of CNN and LSTM (41) with the CHB-MIT dataset. Therefore, we must perform single-channel seizure detection using other deep-learning techniques and examine their computational complexity. Moreover, we need to carry out feature analysis studies based on explainable deep learning methods to understand possible reasons for falsely detected and missed seizures to improve our single-channel seizure detection performance. Third, we empirically set the post-processing parameters in the event-level evaluation to maximize the seizure detection performance for each patient. As a trade-off exists between sensitivity and FAR, the parameters should be carefully tuned for each patient to avoid erroneous detection as much as possible. Therefore, we must consider more systematic designs to optimize the parameters, such as adaptive thresholding, to reflect seizure patterns and tunable artifact rejection, and reduce excessive false alarms (55–57). Finally, to strengthen our findings and to validate our approaches’ effectiveness, we must increase the number of patients through other open datasets or collaborative multicenter studies beyond the 13 patients in the CHB-MIT dataset. However, we need to carefully concern that larger studies demand higher time and cost consumption.

## 5 Conclusion

We constructed patient-specific deep learning-based 18-, 4-, and single-channel automated seizure detectors using long-term scalp EEG recordings from the CHB-MIT dataset by reducing the number of EEG channels. Note that neurologists selected a specific channel based on the spatial seizure characteristics of individual patients among the four channels in which two were close to the behind-the-ear regions, and the other two were at the frontal lobe for our single-channel detectors. The seizure detection performance of our single-channel detectors achieved an average sensitivity of >99% and FAR of <0.3/h, which were comparable to those of our multi-channel detectors. In addition, the re-annotation of well-characterized seizures could partially improve the performance of single-channel seizure detection, particularly for false alarms and latency. We suggest that our single-channel approach in conjunction with neurologists’ clinical designation of the most prominent seizure locations has a high potential for application in wearable devices for seizure detection in long-term EEG recordings for patients with refractory epilepsy.

## Data availability statement

Publicly available datasets were analyzed in this study. This data can be found here: the Children’s Hospital Boston-Massachusetts Institute of Technology (CHB-MIT) scalp EEG database repository, https://physionet.org/content/chbmit/1.0.0/.

## Ethics statement

The study involving humans was approved by the Institutional Review Board of Seoul National University Bundang Hospital (No. B-2205-758-105). The study was conducted in accordance with the local legislation and institutional requirements. The ethics committee/institutional review board waived the requirement of written informed consent for participation from the participants or the participants’ legal guardians/next of kin because of the retrospective nature of the study.

## Author contributions

YC: Data curation, Formal analysis, Methodology, Software, Validation, Visualization, Writing – original draft, Writing – review & editing. AC: Conceptualization, Data curation, Investigation, Methodology, Visualization, Writing – review & editing. HK: Conceptualization, Data curation, Formal analysis, Funding acquisition, Investigation, Methodology, Project administration, Resources, Supervision, Validation, Visualization, Writing – original draft, Writing – review & editing. KK: Conceptualization, Investigation, Methodology, Writing – review & editing.

## Footnotes

^1^ https://physionet.org/content/chbmit/1.0.0/
