# Supplementary material for: Single-channel seizure detection with clinical confirmation of seizure locations using CHB-MIT dataset
Source: Front Neurol. 2024 May 20;15:1389731. doi: 10.3389/fneur.2024.1389731 (PMC11148866; doi:10.3389/fneur.2024.1389731)
Supplement: Supplementary file 1 [file Data_Sheet_1.docx]

Supplementary Material

# Supplementary Tables

**Supplementary Table 1.** Performance of patient-specific 18-channel seizure detectors for ictal-interictal classification (segment-level) and seizure detection (event-level). Segment-level evaluation represents mean ± standard deviation (SD) for *k*-fold cross-validation. Latency of event-level evaluation represents mean ± SD over all seizures in each case. AUC and FAR denote area under the receiver operating characteristic curve and false alarm rate, respectively.

|  | Segment-level evaluation | | | | Event-level evaluation | | | | | | |
| --- | --- | --- | --- | --- | --- | --- | --- | --- | --- | --- | --- |
| Case | Sensitivity (%) | Specificity (%) | Accuracy (%) | AUC  (%) | Sensitivity (%) | FAR  (/h) | Latency  (s) | EEG len. for evaluation  (h) | Correctly detected seizure | Missed seizure | False  alarm |
| chb01 | 99.89±0.30 | 99.76±0.26 | 99.76±0.26 | 99.82±0.17 | 100.00 | 0.00 | 5.4±6.1 | 6.6 | 7 | 0 | 0 |
| chb02 | 98.12±3.25 | 99.97±0.03 | 99.97±0.03 | 99.04±1.61 | 100.00 | 0.00 | 2.3±4.7 | 2.3 | 3 | 0 | 0 |
| chb03 | 98.99±1.25 | 96.34±1.71 | 96.34±1.71 | 97.67±0.59 | 100.00 | 0.29 | 9.6±5.3 | 7.0 | 7 | 0 | 2 |
| chb04 | 98.18±2.52 | 99.97±0.02 | 99.97±0.02 | 99.08±1.26 | 100.00 | 0.00 | 0.8±2.1 | 10.7 | 4 | 0 | 0 |
| chb05 | 99.23±1.73 | 99.97±0.02 | 99.96±0.02 | 99.59±0.86 | 100.00 | 0.00 | 11.2±18.4 | 5.0 | 5 | 0 | 0 |
| chb07 | 100.00±0.00 | 99.82±0.11 | 99.82±0.11 | 99.91±0.06 | 100.00 | 0.11 | 12.0±10.8 | 9.0 | 3 | 0 | 1 |
| chb08 | 99.21±1.36 | 99.91±0.08 | 99.90±0.08 | 99.56±0.65 | 100.00 | 0.40 | 2.2±1.9 | 5.0 | 5 | 0 | 2 |
| chb10 | 98.77±2.62 | 98.91±1.43 | 98.91±1.43 | 98.84±1.21 | 100.00 | 0.36 | -3.4±6.3 | 14.0 | 7 | 0 | 5 |
| chb11 | 98.95±1.81 | 99.90±0.08 | 99.90±0.08 | 99.42±0.88 | 100.00 | 1.43 | -2.3±1.5 | 2.8 | 3 | 0 | 4 |
| chb15 | 96.20±5.32 | 90.24±9.13 | 90.33±8.93 | 93.23±3.78 | 100.00 | 1.14 | -11.8±17.1 | 14.0 | 20 | 0 | 16 |
| chb17 | 96.59±4.61 | 97.73±1.66 | 97.72±1.65 | 97.16±1.52 | 100.00 | 0.00 | -2.0±6.6 | 3.0 | 3 | 0 | 0 |
| chb22 | 100.00±0.00 | 99.39±0.24 | 99.39±0.24 | 99.69±0.12 | 100.00 | 0.00 | 6.0±3.0 | 3.0 | 3 | 0 | 0 |
| chb23 | 98.42±2.74 | 98.17±1.41 | 98.17±1.40 | 98.29±1.03 | 100.00 | 0.11 | -2.1±8.4 | 9.0 | 7 | 0 | 1 |
| Mean± SD | 98.66±1.19 | 98.47±2.71 | 98.47±2.69 | 98.56±1.81 | 100.00±0.00 | 0.30±0.47 | 2.1±6.7 | 7.0±4.1 | 5.9±4.6 | 0.0±0.0 | 2.4±4.4 |

**Supplementary Table 2.** Performance of patient-specific 4-channel seizure detectors for ictal-interictal classification (segment-level) and seizure detection (event-level). Segment-level evaluation represents mean ± standard deviation (SD) for *k*-fold cross-validation. Latency of event-level evaluation represents mean ± SD over all seizures in each case. AUC and FAR denote area under the receiver operating characteristic curve and false alarm rate, respectively.

|  | Segment-level evaluation | | | | Event-level evaluation | | | | | | |
| --- | --- | --- | --- | --- | --- | --- | --- | --- | --- | --- | --- |
| Case | Sensitivity  (%) | Specificity (%) | Accuracy (%) | AUC  (%) | Sensitivity (%) | FAR  (/h) | Latency  (s) | EEG len. for evaluation (h) | Correctly detected seizure | Missed seizure | False  alarm |
| chb01 | 99.55±0.62 | 99.78±0.22 | 99.77±0.22 | 99.66±0.28 | 100.00 | 0.00 | 5.7±6.4 | 6.6 | 7 | 0 | 0 |
| chb02 | 89.07±18.94 | 99.78±0.19 | 99.77±0.17 | 94.42±9.37 | 100.00 | 0.00 | 2.3±6.4 | 2.3 | 3 | 0 | 0 |
| chb03 | 99.89±0.30 | 97.36±2.59 | 97.36±2.58 | 98.62±1.24 | 100.00 | 0.29 | 9.4±6.1 | 7.0 | 7 | 0 | 2 |
| chb04 | 93.75±10.83 | 99.92±0.07 | 99.92±0.07 | 96.83±5.39 | 100.00 | 0.00 | 0.8±1.3 | 10.7 | 4 | 0 | 0 |
| chb05 | 99.69±0.70 | 99.92±0.06 | 99.92±0.06 | 99.80±0.33 | 100.00 | 0.00 | 4.4±1.1 | 5.0 | 5 | 0 | 0 |
| chb07 | 97.66±3.37 | 99.27±1.19 | 99.27±1.18 | 98.46±1.40 | 100.00 | 0.00 | 8.0±3.6 | 9.0 | 3 | 0 | 0 |
| chb08 | 100.00±0.00 | 99.74±0.20 | 99.74±0.20 | 99.87±0.10 | 100.00 | 0.60 | 2.6±0.5 | 5.0 | 5 | 0 | 3 |
| chb10 | 99.66±0.63 | 99.47±0.73 | 99.47±0.73 | 99.56±0.36 | 100.00 | 0.29 | -0.4±6.3 | 14.0 | 7 | 0 | 4 |
| chb11 | 100.00±0.00 | 99.27±1.10 | 99.27±1.10 | 99.63±0.55 | 100.00 | 2.51 | 9.3±17.9 | 2.8 | 3 | 0 | 7 |
| chb15 | 92.80±9.47 | 87.86±10.79 | 87.93±10.51 | 90.33±3.49 | 95.00 | 1.57 | -7.4±14.2 | 14.0 | 19 | 1 | 22 |
| chb17 | 92.95±9.05 | 95.74±6.81 | 95.73±6.78 | 94.35±3.22 | 66.67 | 0.00 | 6.0±11.3 | 3.0 | 2 | 1 | 0 |
| chb22 | 100.00±0.00 | 99.32±0.38 | 99.32±0.38 | 99.66±0.20 | 100.00 | 0.00 | 6.3±3.1 | 3.0 | 3 | 0 | 0 |
| chb23 | 100.00±0.00 | 92.97±3.38 | 93.00±3.36 | 96.48±1.69 | 100.00 | 0.00 | -1.0±7.9 | 9.0 | 7 | 0 | 0 |
| Mean± SD | 97.31±3.78 | 97.72±3.61 | 97.73±3.59 | 97.51±2.94 | 97.05±9.23 | 0.40±0.77 | 3.5±4.8 | 7.0±4.1 | 5.8±4.4 | 0.2±0.4 | 2.9±6.1 |

**Supplementary Table 3.** Performance of patient-specific single-channel seizure detectors for ictal-interictal classification (segment-level) and seizure detection (event-level). Segment-level evaluation represents mean ± standard deviation (SD) for *k*-fold cross-validation. Latency of event-level evaluation represents mean ± SD over all seizures in each case. AUC and FAR denote area under the receiver operating characteristic curve and false alarm rate, respectively.

|  |  | Segment-level evaluation | | | | Event-level evaluation | | | | | | |
| --- | --- | --- | --- | --- | --- | --- | --- | --- | --- | --- | --- | --- |
| Case | Single-channel | Sensitivity (%) | Specificity (%) | Accuracy (%) | AUC  (%) | Sensitivity (%) | FAR  (/h) | Latency (s) | EEG len. for evaluation (h) | Correctly detected seizure | Missed seizure | False alarm |
| chb01 | P8-O2 | 95.53±9.22 | 97.68±2.52 | 97.68±2.50 | 96.61±4.17 | 100.00 | 0.15 | 3.3±4.2 | 6.6 | 7 | 0 | 1 |
| chb02 | P7-O1 | 99.75±0.44 | 99.94±0.02 | 99.94±0.02 | 99.84±0.21 | 100.00 | 0.88 | 0.7±4.0 | 2.3 | 3 | 0 | 2 |
| chb03 | Fp1-F3 | 99.11±2.36 | 96.80±2.31 | 96.81±2.30 | 97.95±1.15 | 100.00 | 0.00 | 9.0±4.9 | 7.0 | 7 | 0 | 0 |
| chb04 | P8-O2 | 96.61±5.87 | 99.27±1.06 | 99.27±1.06 | 97.94±2.70 | 100.00 | 0.00 | 1.0±1.4 | 10.7 | 4 | 0 | 0 |
| chb05 | P7-O1 | 97.65±3.59 | 99.21±0.72 | 99.21±0.71 | 98.43±1.56 | 100.00 | 0.00 | 12.4±19.4 | 5.0 | 5 | 0 | 0 |
| chb07 | Fp1-F3 | 100.00±0.00 | 99.75±0.18 | 99.75±0.18 | 99.87±0.09 | 100.00 | 0.11 | 11.7±10.0 | 9.0 | 3 | 0 | 1 |
| chb08 | Fp1-F3 | 99.37±1.40 | 97.50±3.09 | 97.50±3.08 | 98.43±1.48 | 100.00 | 0.80 | 4.2±7.3 | 5.0 | 5 | 0 | 4 |
| chb10 | P7-O1 | 99.10±2.04 | 99.93±0.05 | 99.93±0.05 | 99.52±1.01 | 100.00 | 0.14 | -1.6±6.4 | 14.0 | 7 | 0 | 2 |
| chb11 | P7-O1 | 100.00±0.00 | 99.94±0.02 | 99.94±0.02 | 99.97±0.01 | 100.00 | 0.72 | 2.3±1.2 | 2.8 | 3 | 0 | 2 |
| chb15 | P7-O1 | 94.47±7.37 | 96.46±3.64 | 96.43±3.53 | 95.46±3.19 | 95.00 | 0.07 | -1.2±8.9 | 14.0 | 19 | 1 | 1 |
| chb17 | P8-O2 | 90.11±14.49 | 96.93±2.76 | 96.91±2.72 | 93.52±6.23 | 100.00 | 0.00 | -6.3±7.1 | 3.0 | 3 | 0 | 0 |
| chb22 | Fp1-F3 | 98.71±2.24 | 99.08±0.46 | 99.08±0.45 | 98.89±0.89 | 100.00 | 0.00 | 7.3±1.2 | 3.0 | 3 | 0 | 0 |
| chb23 | Fp1-F3 | 87.49±21.67 | 93.96±8.61 | 93.92±8.54 | 90.72±8.93 | 100.00 | 0.00 | -0.3±7.7 | 9.0 | 7 | 0 | 0 |
| Mean± SD |  | 96.76±3.97 | 98.19±1.82 | 98.18±1.83 | 97.47±2.77 | 99.62±1.39 | 0.22±0.34 | 3.3±5.5 | 7.0±4.1 | 5.8±4.3 | 0.1±0.3 | 1.0±1.2 |

**Supplementary Table 4.** Performance of patient-specific single-channel seizure detectors trained by publicly available annotations for ictal-interictal classification (segment-level) and seizure detection (event-level). Segment-level evaluation represents mean ± standard deviation (SD) for *k*-fold cross-validation. Latency of event-level evaluation represents mean ± SD over all seizures in each case. AUC and FAR denote area under the receiver operating characteristic curve and false alarm rate, respectively.

|  |  | Segment-level evaluation | | | | Event-level evaluation | | | | | | |
| --- | --- | --- | --- | --- | --- | --- | --- | --- | --- | --- | --- | --- |
| Case | Single-channel | Sensitivity (%) | Specificity (%) | Accuracy (%) | AUC  (%) | Sensitivity (%) | FAR  (/h) | Latency (s) | EEG len. for evaluation (h) | Correctly detected seizure | Missed seizure | False alarm |
| chb01 | P8-O2 | 95.98±5.83 | 96.92±2.83 | 96.92±2.81 | 96.45±2.18 | 100.00 | 0.15 | 5.3±3.8 | 6.6 | 7 | 0 | 1 |
| chb02 | P7-O1 | 100.00±0.00 | 99.84±0.12 | 99.84±0.12 | 99.92±0.06 | 100.00 | 0.88 | 0.0±4.4 | 2.3 | 3 | 0 | 2 |
| chb03 | Fp1-F3 | 99.10±1.15 | 98.39±0.59 | 98.40±0.58 | 98.75±0.44 | 100.00 | 0.00 | 8.6±4.9 | 7.0 | 7 | 0 | 0 |
| chb04 | P8-O2 | 92.62±4.95 | 99.85±0.24 | 99.83±0.22 | 95.80±1.96 | 75.00 | 0.09 | 7.0±8.7 | 10.7 | 3 | 1 | 1 |
| chb05 | P7-O1 | 94.99±3.61 | 95.33±7.94 | 95.32±7.91 | 95.16±3.02 | 100.00 | 0.00 | 15.4±14.2 | 5.0 | 5 | 0 | 0 |
| chb07 | Fp1-F3 | 97.91±1.97 | 97.57±2.56 | 97.57±2.56 | 97.74±2.19 | 100.00 | 0.00 | 22.7±26.3 | 9.0 | 3 | 0 | 0 |
| chb08 | Fp1-F3 | 92.34±3.05 | 88.40±7.37 | 88.44±7.24 | 90.37±2.29 | 100.00 | 0.20 | 28.4±33.2 | 5.0 | 5 | 0 | 1 |
| chb10 | P7-O1 | 97.99±2.79 | 99.32±1.03 | 99.32±1.03 | 98.65±1.29 | 100.00 | 0.29 | 1.7±4.9 | 14.0 | 7 | 0 | 4 |
| chb11 | P7-O1 | 98.69±1.20 | 99.79±0.15 | 99.78±0.15 | 99.24±0.64 | 100.00 | 0.00 | 3.3±6.7 | 2.8 | 3 | 0 | 0 |
| chb15 | P7-O1 | 94.47±6.78 | 89.87±7.11 | 89.96±6.89 | 92.17±3.08 | 95.00 | 0.50 | 4.4±9.2 | 14.0 | 19 | 1 | 7 |
| chb17 | P8-O2 | 100.00±0.00 | 70.01±10.88 | 70.10±10.86 | 85.00±5.44 | 100.00 | 0.00 | -5.3±7.6 | 3.0 | 3 | 0 | 0 |
| chb22 | Fp1-F3 | 93.19±5.94 | 99.57±0.30 | 99.56±0.29 | 96.38±2.82 | 100.00 | 0.00 | 10.7±3.8 | 3.0 | 3 | 0 | 0 |
| chb23 | Fp1-F3 | 95.83±2.96 | 99.05±0.57 | 99.04±0.56 | 97.44±1.20 | 100.00 | 0.00 | 1.3±2.6 | 9.0 | 7 | 0 | 0 |
| Mean± SD |  | 96.39±2.75 | 94.92±8.38 | 94.93±8.35 | 95.62±4.20 | 97.69±6.96 | 0.16±0.26 | 8.0±9.4 | 7.0±4.1 | 5.8±4.4 | 0.2±0.4 | 1.2±2.1 |

**Supplementary Table 5.** Channel selection methods of previous studies using the CHB-MIT dataset in comparison with our single-channel seizure detection approach. LSTM, SVM, RUSBoost, and CNN denote long short-term memory, support vector machine, random undersampling and boosting, and convolutional neural network, respectively.

| Study | Number of channels | Channel selection method | | Classification method |
| --- | --- | --- | --- | --- |
| Y. Song et al. | Not specified | Automated (Mathematical approach) | Select channels using the refine composite multiscale dispersion entropy to measure EEG complexity | Residual convolutional LSTM |
| F. -G. Tang et al. | 5 | Automated (Mathematical approach) | Select channels that correspond to the highest variance difference between ictal and interictal states | Autoencoder (for feature extraction), SVM |
| S. Khanmohammadi et al. | 5 | Automated (Mathematical approach) | Select channels that correspond to the highest variance difference between ictal and interictal states | Adaptive distance-based change point detector |
| R. Asif et al. | 6, 8, 10, and 12 | Manual (Considering the temporal lobe epilepsy) | Select channels that correspond to the left and right temporal ones | RUSBoost |
| **This study (Single-channel)** | 1 | Manual (Neurologist's confirmation of seizure locations) | Select one of four channels correspond to the seizure location confirmed by neurologists | Stacked 2D-CNN |

**Supplementary Table 6.** Single-channel seizure detection performance of chb11 in accordance with four channels (event-level evaluation, total number of seizures = 3, total EEG length = 2.8 h). P7-O1 is the most appropriate channel confirmed by neurologists. FAR denotes false alarm rate. Latency represents mean ± standard deviation over all seizures in chb11.

| Channel | Sensitivity (%) | FAR  (/h) | Latency  (s) | Correctly detected seizure | Missed seizure | False  alarm |
| --- | --- | --- | --- | --- | --- | --- |
| Fp1-F3 | 100.00 | 0.72 | -3.7±3.8 | 3 | 0 | 2 |
| Fp2-F4 | 66.67 | 2.15 | 11.0±24.0 | 2 | 0 | 6 |
| **P7-O1** | **100.00** | **0.36** | **1.7±2.3** | **3** | **0** | **1** |
| P8-O2 | 66.67 | 2.15 | 28.0±49.5 | 2 | 0 | 6 |
